# Scalable and High-Throughput In Vitro Vibratory Platform for Vocal Fold Tissue Engineering Applications

**DOI:** 10.3390/bioengineering10050602

**Published:** 2023-05-17

**Authors:** Andreea Biehl, Ramair Colmon, Anastasia Timofeeva, Ana Maria Gracioso Martins, Gregory R. Dion, Kara Peters, Donald O. Freytes

**Affiliations:** 1 Joint Department of Biomedical Engineering, North Carolina State University & University of North Carolina-Chapel Hill, 4130 Engineering Building III, Campus Box 7115, Raleigh, NC 27695, USArcolmon@ncsu.edu (R.C.); agracio@ncsu.edu (A.M.G.M.); 2 Comparative Medicine Institute, North Carolina State University, 1060 William Moore Drive, Raleigh, NC 27606, USA; 3 Department of Mechanical and Aerospace Engineering, North Carolina State University, Raleigh, NC 27695, USA; aatimofe@ncsu.edu (A.T.); kjpeters@ncsu.edu (K.P.); 4 Department of Otolaryngology-Head and Neck Surgery, University of Cincinnati, Cincinnati, OH 45267, USA; diongy@ucmail.uc.edu

**Keywords:** vocal fold, bioreactor, piezoelectric speaker, vibration, frequency, displacement, fibroblasts, mesenchymal stem cells, gene expression

## Abstract

The vocal folds (VFs) are constantly exposed to mechanical stimulation leading to changes in biomechanical properties, structure, and composition. The development of long-term strategies for VF treatment depends on the characterization of related cells, biomaterials, or engineered tissues in a controlled mechanical environment. Our aim was to design, develop, and characterize a scalable and high-throughput platform that mimics the mechanical microenvironment of the VFs in vitro. The platform consists of a 24-well plate fitted with a flexible membrane atop a waveguide equipped with piezoelectric speakers which allows for cells to be exposed to various phonatory stimuli. The displacements of the flexible membrane were characterized via Laser Doppler Vibrometry (LDV). Human VF fibroblasts and mesenchymal stem cells were seeded, exposed to various vibratory regimes, and the expression of pro-fibrotic and pro-inflammatory genes was analyzed. Compared to current bioreactor designs, the platform developed in this study can incorporate commercial assay formats ranging from 6- to 96-well plates which represents a significant improvement in scalability. This platform is modular and allows for tunable frequency regimes.

## 1. Introduction

Voice is produced by the vibration of the vocal folds (VFs), unique multi-layered structures comprising a stratified squamous epithelium, a basement membrane, the lamina propria (LP), and the thyroarytenoid (or vocalis) muscle. Collectively, this multi-layered structure allows for vibration and, ultimately, phonation. The LP is a flexible vibratory structure composed of three layers (superficial LP, intermediate LP, deep LP) that plays a critical role in mediating phonation and is made up of a specialized extracellular matrix comprising glycosaminoglycans, collagen, and elastic fibers. During phonation, the human VFs have the ability to oscillate with amplitudes of 0.1 to 0.5 mm while sustaining frequencies up to 8000 Hertz (Hz) [1,2]; however, as reported by Miri et al., VFs typically vibrate at frequency ranges of 60–200 Hz in males and 160–300 Hz in females [2]. As a result of the vibration necessary to phonate, the LP is exposed to multiple complex mechanical forces including tensile, contractile, aerodynamic, inertial, collision, and shear [3] as well as numerous external factors that can lead to inflammation and subsequent microstructure damage ultimately disrupting vibratory function.

Voice quality can be affected by various external factors such as voice overuse or misuse [4,5,6,7], chemical exposure due to smoke inhalation [8,9], intubation [10,11,12,13], traumas [14,15,16], radiation [17], gastroesophageal reflux [18,19,20,21,22,23], sleeping disorders and aging [24], etc. Abnormalities in pitch, tone, volume, vocal fatigue, voice breaks, or other vocal qualities may be indicative of voice disorders which are estimated to affect approximately 28 million Americans [25]. Vocal pathologies leading to dysphonia include vocal fold cysts, ulcers, scarring/fibrosis, lesions, polyps, nodules, vocal fold cancer, vocal fold paralysis, etc. Current treatment strategies for voice disorders include voice therapy to improve vocal efficiency and reduce pressure on the VFs, phonomicrosurgery (e.g., laser surgery for cancer removal, biopsy of throat lesions, laryngoplasty, laryngeal reinnervation etc.), and local corticosteroid therapy for benign VF lesions. Additionally, advances in biomaterials and cell therapy research have led to novel treatments for voice disorders such as mesenchymal stem cell (MSC) transplants and injection augmentation using bulking agents (e.g., collagen, hyaluronic acid, carboxymethylcellulose, calcium hydroxyapatite, micronized dermis, autologous fat, etc.). Although these treatments have shown promise, none has fully satisfied the regeneration of injured VFs [26]. Recent attempts at bioengineered vocal fold mucosa show promise but are not yet ready for human implantation [27].

The development, maintenance, and regeneration following injury of the VFs are heavily influenced by the phonation-induced mechanical stimulation [25,28]. As a result, various in vitro systems have been developed to closely mimic the mechanical environment of the VFs while incorporating relevant biomaterials, biochemical cues, and cells in a controlled experimental manner. Over the past 20 years, actuator, speaker, rheometer, vacuum, and airflow based bioreactors have been designed and tested to enable faster, more controlled, and affordable characterization of potential VF therapies. However, there is a lack of established parameters for oscillatory regimes (frequency, amplitude, periodicity, exposure time), mechanical forces applied to the system, type and number of cells, biomaterials/substrates used, and 2D vs. 3D cell cultures. It is important to note that for large scale studies (e.g., drug response testing, disease modeling etc.) the total number of experimental units that a bioreactor setting can allow should be considered. In the review by Martins et al. [26], the authors have identified that existing bioreactors have a relatively low number of experimental units; the highest number was identified for the system developed by Bartlett et al. [29] with 16 units.

**Figure 1:**
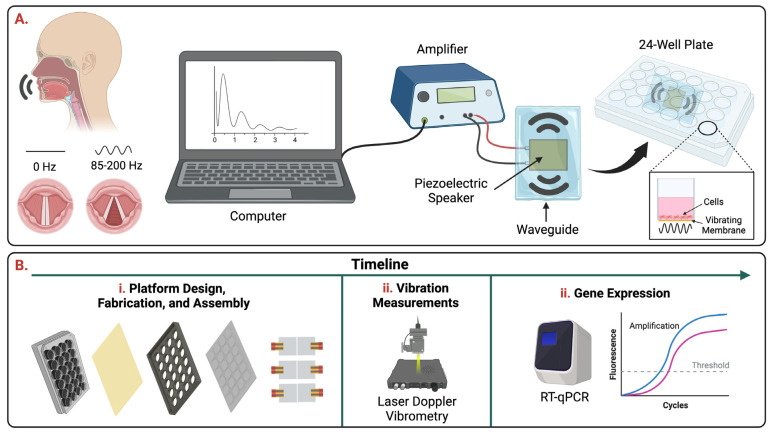
Overview. (**A**) Schematic of the in vitro vibratory platform in which a piezoelectric speaker is attached to a polydimethylsiloxane (PDMS) waveguide that allows the waves to be propagated to a cell culture plate. The speaker is connected to an amplifier which can be controlled via a computer to input the frequency and sound volume. (**B**) Timeline of experiments.

The goal of this study was to design, fabricate, and characterize a high-throughput, easy to construct, and affordable platform that simulates the VF microenvironment in vitro (Figure 1). The platform described in this study is composed of a commercially available bottomless 24-well plate fitted with a flexible membrane atop a custom-designed waveguide equipped with a set of piezoelectric speakers for micron-scale vibrations. In this study we focus on demonstrating that a digitally controlled piezoelectric speaker can produce micron-scaled vibrations that propagate into cell culture via a waveguide and simulate displacements and frequencies of the order of those found during human phonation. The work reported here is the first scalable high-throughput in vitro vibratory platform that can incorporate commercial assay formats ranging from 6- to 96-well plates.

## 2. Materials and Methods

### 2.1. Platform Fabrication and Assembly

**Figure 2:**
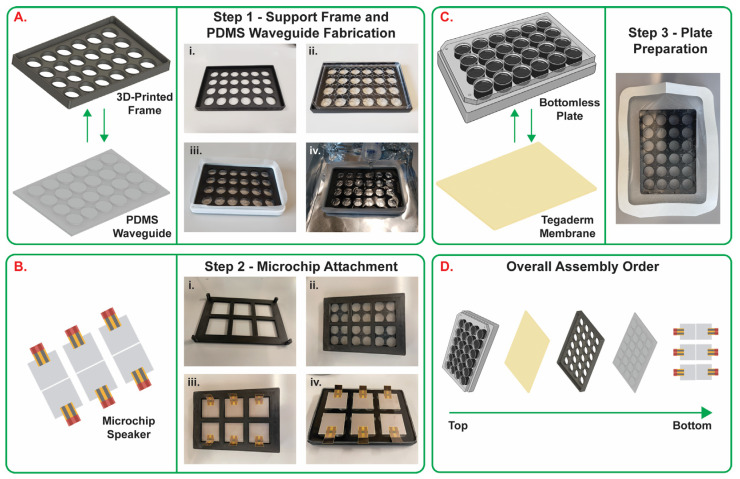
Platform fabrication. (**A**) Step 1: support frame and PDMS waveguide: (i) real-life image of the 3D printed support frame, (ii) a well plate lid is placed on the 3D printed frame to prevent leaking, (iii) a foam and tape wall is placed on the side of the frame, (iv) PDMS is poured and cured for 4 days. (**B**) Step 2: piezoelectric speaker placement: (i) a 3D printed frame is (ii) placed on the PDMS waveguide, and (iii) six piezoelectric speakers are placed equidistantly. (iv) Top view of the assembled PDMS waveguide with attached piezoelectric speakers. (**C**) Step 3: cell culture plate preparation: the Tegaderm^TM^ flexible membrane is attached to the bottom of a 24-well bottomless plate. (**D**) Overall assembly order of each component from Steps 1–3.

The in vitro vibratory platform consisted of the following components: (1) a bottomless 24-well plate (VWR, Radnor, PA, USA), (2) a Tegaderm^TM^ semi-transparent film dressing (3M, Saint Paul, MN, USA) acting as a flexible membrane, (3) a frame designed in Autodesk Inventor and 3D printed from Xometry (Gaithersburg, MD, USA), (4) a polydimethylsiloxane (PDMS) waveguide, and (5) six piezoelectric speakers (445-181632-ND; Digi-Key Electronics, Thief River Falls, MN, USA) (Figure 2). The PDMS waveguide (Figure 2A) was fabricated using 50 mL of PDMS with a 19:1 base to crosslinking agent ratio. The 3D printed support frame was designed so that the PDMS waveguide would have 24 equidistant holes with a height of 8 mm and radius of 8 mm each. The PDMS was poured onto the bottom of the support frame with the lid of a 24-well plate inserted into the top side of the frame. A wall of foam and tape was placed at the bottom and on the sides of the support frame to prevent leaking and maintain consistent bottom to top dimensions. The PDMS was set to cure for 4 days at room temperature on a level surface. The PDMS waveguide was then integrated with 6 piezoelectric speakers equidistant from each other. Spatial positioning of the piezoelectric speakers was facilitated using a positioning frame designed in Autodesk Inventor and printed with a Lulzbot Mini 3D printer (LulzBot, Fargo, ND, USA) to ensure that the speakers were evenly spaced (Figure 2B). Double-sided tape (Nitto Denko, Osaka, Japan) was used to adhere the piezoelectric speakers to the waveguide. The positioning frame was removed after the piezoelectric speakers were attached. Finally, the Tegaderm^TM^ adhesive dressing was placed to cover the bottom of the bottomless 24-well plate (Figure 2C) which was then placed on the PDMS waveguide with attached piezoelectric speakers (Figure 2D).

**Figure 3:**
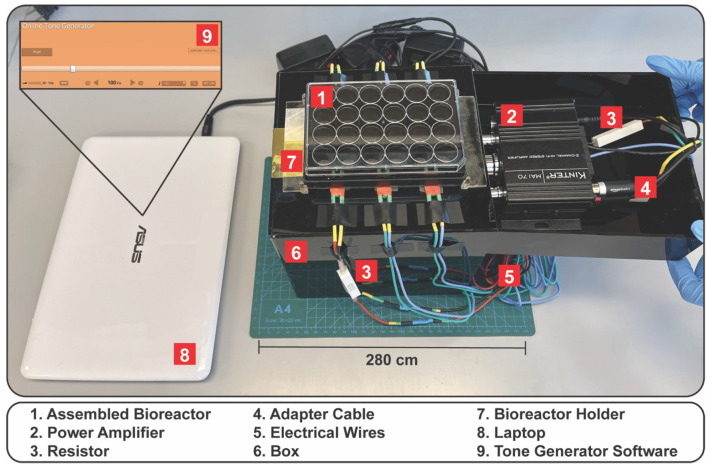
Fully assembled system placed on the benchtop.

**Figure 4:**
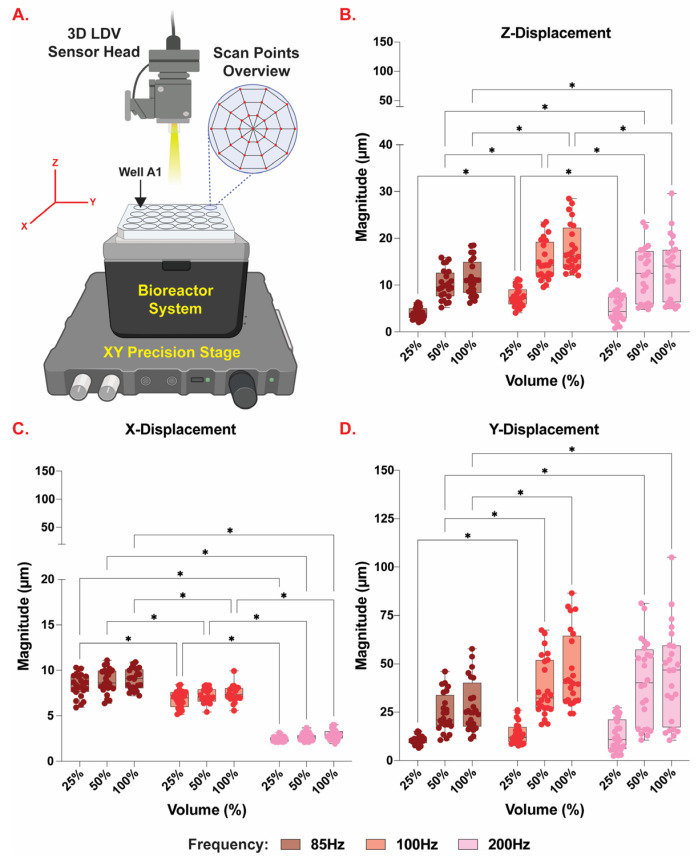
Average axial Tegaderm^TM^ dressing displacement in μm with respect to speaker frequency and volume. (**A**) Computer generated schematic of bioreactor positioning on the LDV XY Precision Stage. (**B**) LDV measured displacement of Tegaderm^TM^ dressing in the *Z*-axis. (**C**) LDV measured displacement of Tegaderm^TM^ dressing in the *X*-axis. (**D**) LDV measured displacement of Tegaderm^TM^ dressing in the *Y*-axis. Bar graphs represent Box and Whiskers plots showing all measured points. * = statistically significant (*p* < 0.05).

**Figure 5:**
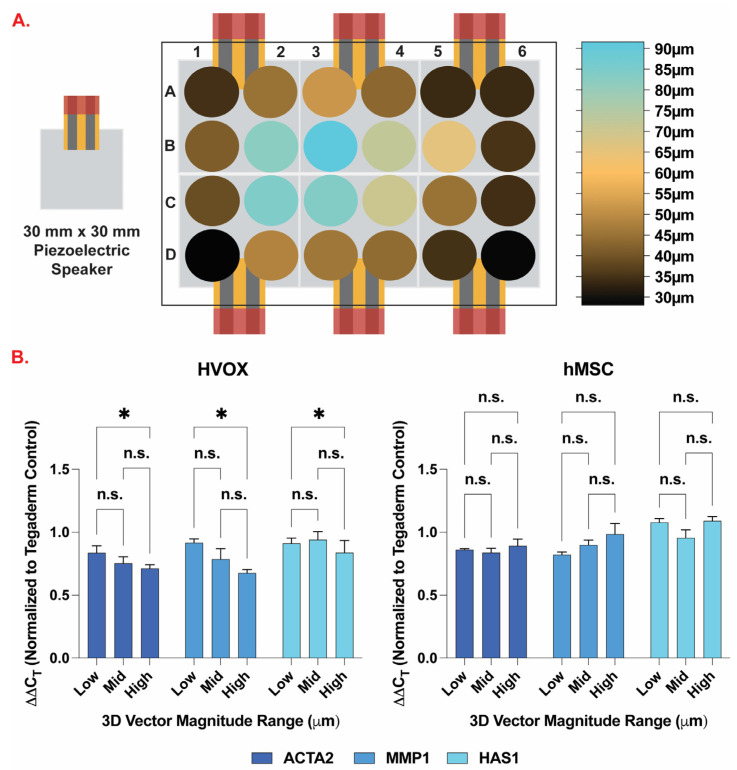
(**A**) Heatmap of LDV measured 3D vector magnitude of Tegaderm^TM^ membrane displacement (µm) vibrated at 100 Hz and 100% sound volume. (**B**) Gene expression profiles for ACTA2, MMP1, and HAS1 for HVOX and hMSCs cultured on top of Tegaderm^TM^ membrane and vibrated at 100 Hz and 100% sound volume for 2 h. Low = 28–35 µm, mid = 42–52 µm, high = 83–92 µm. Bar graphs represent mean ± standard error of the mean (SEM). n = 3 samples analyzed per experimental group. * = statistically significant (*p* < 0.05). n.s. = not significant (*p* > 0.05).

The fully assembled platform (Figure 3-1) was secured using a 3D printed holder (Figure 3-7) onto a box (Figure 3-6) and connected via electrical wires (Figure 3-5) to resistors (A137420-ND; Digi-Key Electronics) (Figure 3-3) and power audio amplifiers (Kinter MA170 12V 2 Channel Mini Digital Audio Power Amplifier; Amazon, Seattle, WA) (Figure 3-2) used to drive the piezoelectric speakers. The amplifiers were connected to a computer (Figure 3-8) via adapter cables (Amazon Basics, Amazon) (Figure 3-4). Tone generator software (https://www.szynalski.com/tone-generator/, accessed on 10 November 2021) (Figure 3-9) was used to control the frequency input. The computer’s sound volume was set to maximum, and the input sound settings for all the experiments performed in this study were further adjusted using the tone generator software to volumes of 25, 50, and 100%. For cell culture experiments, the system was installed in a common CO_2_ incubator, while the computer was located outside the CO_2_ incubator.

### 2.2. Laser Doppler Vibrometry (LDV) Measurements

Frequency and amplitude precision were evaluated using Laser Doppler Vibrometry (LDV) (MSA-100-3D Micro System Analyzer, Polytec, VA) by measuring the displacement of the Tegaderm^TM^ flexible membrane during various oscillatory routines. LDV was also used to characterize the displacement of the piezoelectric speaker in the XY plane and in the Z direction at 100 Hz and 100% sound volumes. Supplementary Figure S1A shows a real-life image of the 3D LDV measurement system used to examine the surface vibration of the flexible membrane with varying input sound frequency and sound volume. The assembled system was placed under the 3D LDV sensor head, on the precision stage. The XYZ coordinate is defined with respect to the coordinate system of the 3D LDV as shown in Figure 4. The XY plane is parallel with the plane of the XY precision stage movement. The Z direction is perpendicular to the XY plane. Prior to measuring the vibration in each well, the surface area of interest was directly spray-coated with a thin layer of white powder (Weld Check^®^ Developer, CRC Industries, Inc., Warminster, PA) to create uniform and sufficient light scattering from the surface of the Tegaderm^TM^ dressing because the flexible membrane is semi-transparent. Vibration of the Tegaderm^TM^ membrane in the bottom of the wells in the assembled bioreactor was scanned at sinusoidal inputs of 85, 100, and 200 Hz frequencies and 25, 50, and 100% sound volumes. Supplementary Figure S1B shows that each LDV scan spatially mapped individual wells using a spider web mesh consisting of 33 nodes per well. The settings used for the 3D LDV are listed in Table 1.

### 2.3. Cell Culture Conditions

A human vocal fold fibroblast cell line (HVOX) produced by the Branski Laboratory was used as previously described [30]. HVOX were cultured in tissue culture plastic (TCP) flasks and maintained in Dulbecco’s Modified Eagle’s Medium (DMEM) (Life Technologies, Carlsbad, CA, USA) with 10% heat-inactivated fetal bovine serum (FBS) (Genesee Scientific) and 1% penicillin/streptomycin. The media were replaced every 2–3 days. HVOX were passaged at 70–80% confluence using 0.25% trypsin–EDTA (Life Technologies) at 37 °C for 5 min, neutralized using maintenance media, and seeded into TCP-treated flasks. HVOX passages 10–15 were used for this study.

Normal human bone marrow derived mesenchymal stem cells (hMSCs) (PT-2501, Lonza, Walkersville, MD, USA) were cultured according to the manufacturer’s instructions. Briefly, hMSCs were cultured in TCP-treated flasks at an initial seeding density of 5000–6000 cells/cm^2^. The maintenance media (MSCBM Basal Medium, PT-3238, Lonza) were replaced every two days. hMSCs were passaged at 70–80% confluence using 0.25% trypsin–EDTA (Life Technologies) at 37 °C for 5 min, neutralized using maintenance media, and seeded into TCP-treated flasks. hMSC passages 5–6 were used for this study.

### 2.4. Gene Expression Analysis

Real-time quantitative polymerase chain reaction (RT-qPCR) was used to analyze the changes in gene expression of pro-fibrotic and pro-inflammatory genes in static cells (control) or cells exposed to vibration. HVOX and hMSC cells were seeded on top of the flexible membrane at a density of 30,000–40,000 cells/well and allowed to attach overnight. The next day, the cells were exposed to a frequency of 100 Hz at 100% sound volume for 1 or 2 h. After a rest period of 6 h, cell samples were collected, lysed using the TRK Lysis Buffer from the E.Z.N.A Total RNA Kit, Omega Bio-Tek (VWR, Radnor, PA), homogenized, and frozen at −80 °C for at least 24 h. Samples were thawed and total RNA was isolated using the E.Z.N.A Total RNA Kit, Omega Bio-Tek (VWR) according to the manufacturer’s instructions. RT-qPCR was performed using the GoTaq^®^ 1-Step RT-qPCR System Kit from Promega (Madison, WI) in QuantStudio 3 (Applied Biosystems, Waltham, MA) using the primers listed in Table 2.

### 2.5. Statistical Analysis

GraphPad Prism 9.0 software was used to perform statistical analyses. All experiments were performed at least three independent times, unless otherwise noted. A value of *p* < 0.05 was considered significant. For LDV data analysis, nodal averaging for each well on the respective directional axes and 3D vector magnitude calculations were performed using Excel and exported to GraphPad Prism 9.0 for further statistical analysis. For gene expression analysis, the results were normalized to GAPDH using Microsoft Excel and exported to GraphPad Prism 9.0 for further statistical analysis. Statistical significance was determined via one-way or two-way ANOVA or using the one sample *t*-test and Wilcoxon test with a theoretical mean of 1.0 representing static culture.

## 3. Results

### 3.1. Vibratory Platform Model

The in vitro vibratory platform (Figure 2 and Figure 3) features six piezoelectric speakers attached to a PDMS waveguide that allows the waves to be propagated to a bottomless twenty-four-well plate equipped with a Tegaderm^TM^ dressing, which acts as a flexible membrane to transmit vibrations and allows for culturing of cells. The PDMS waveguide was 4 mm thick with 24 equidistant circles that fit under each well of the bottomless 24-well plate. The piezoelectric speakers are connected to amplifiers which can be controlled via a computer. The frequencies of vibrational signals used in this study were 85, 100, and 200 Hz. This range incorporates values around the mean male and female fundamental frequencies of 112.0 Hz and 195.8 Hz [31]. While not including the lowest and highest frequency values commonly found in males (60–200 Hz) and females (160–300 Hz), the frequency values tested in this study were in the range of regular speech fundamental frequencies for both males and females.

### 3.2. Platform Characterization

Laser Doppler Vibrometry (LDV) was used to measure the displacement of the Tegaderm^TM^ flexible membrane during different oscillatory routines (85, 100, and 200 Hz frequency at 25, 50, and 100% sound volume). Figure 4 shows the displacement in the X, Y, and Z directions measured from each individual well of the 24-well plate. A total of 33 locations, as indicated by the spider mesh in Figure 4A, were sampled for each well and averaged together. For the X direction, the different sound volume regimes did not significantly affect displacement in any of the frequencies applied (*p* > 0.05). In the Y direction, there was no difference in displacement between 85 Hz and 200 Hz or 100 Hz and 200 Hz at 25% volume, 100 Hz and 200 Hz at 50% volume, and 100 Hz and 200 Hz at 100% volume (*p* > 0.05). For the Z direction, the change in frequency from 85 Hz to 200 Hz at 25% volume did not significantly alter the displacement of the Tegaderm^TM^ dressing (*p* > 0.05). Supplementary Figure S2 shows the displacement of the piezoelectric speakers in the X, Y, and Z directions, respectively, given the sinusoidal oscillatory routine of 100 Hz and 100% sound volume. A total of 441 equidistant locations were sampled for the entire surface of the piezoelectric speaker. There were significant differences among the amplitudes of displacement in each coordinate axis; with the greatest displacement in the Z direction and the lowest in the X direction (*p* > 0.05).

### 3.3. Gene Expression

Using RT-qPCR, we assessed the effect of vibratory regime (100 Hz, 100% sound volume) on the relative expression of three pro-fibrotic and pro-inflammatory genes: smooth muscle alpha-2 actin (ACTA2), matrix metalloproteinase 1 (MMP1), and hyaluronan synthase 1 (HAS1) (Figure 5). Three displacement ranges were analyzed in this study: low = 28–35 µm, mid = 42–52 µm, high = 83–92 µm. These ranges were selected based on data analysis shown in Figure 5A. The 3D vector magnitude of each well was calculated using the mathematical formula for the distance between two points in space (Equation (1)). The outside 16 wells in the well plate fell within the low displacement category. The majority of the wells that make up the inner eight wells, however, experienced mid and high displacement regimes with the exception of well C5.

(1) $$V\;=\sqrt{\left({\left(x_{2}-x_{1}\right)}^{2}+{\left(y_{2}-y_{1}\right)}^{2}+{\left(z_{2}-z_{1}\right)}^{2}\right)}$$

ACTA2, MMP1, and HAS1 expression in HVOX was significantly lower at the high displacement range compared to the low displacement range (*p* < 0.05). No significant changes were detected between low and mid or mid and high displacement ranges at 2 h of exposure. As shown in Supplementary Figure S3, ACTA2 expression in HVOX exposed to vibration was significantly lower compared to static control in the following conditions: mid range at 2 h of exposure, high range at 1 and 2 h of exposure. For MMP1, only the high range was significantly lower than static control at both 1 and 2 h of exposure. No significant changes were detected in HAS1 expression for any of the conditions.

In hMSCs, no significant changes were detected between any of the displacement ranges. When comparing to static control (Supplementary Figure S3), significant changes were detected as following: (1) ACTA2 expression was significantly lower for the low range at 2 h of exposure, mid range at 1 and 2 h of exposure, and high range at 1 h of exposure; (2) MMP1 expression was significantly lower for the low range at 2 h of exposure; (3) no significant changes were detected in HAS1 expression for any of the conditions.

## 4. Discussion

Mechanical stimulation plays an important role in the development, maintenance, and regeneration of the vocal folds following injury. As a result, it is important to develop bioreactors/platforms that replicate the phonatory conditions of the vocal folds in vitro which are commonly defined as amplitudes ranging from 0.1 to 1.0 mm and frequencies of 60 to 300 Hz [1,2]. Our group designed and characterized a vibratory platform that can generate frequencies resembling human phonation (Figure 1). The design comprises a commercially available twenty-four-well bottomless plate fitted with a flexible membrane atop a custom-designed PDMS waveguide equipped with six piezoelectric speakers (Figure 2 and Figure 3). The platform was capable of generating frequencies of 85, 100, and 200 Hz. However, the piezoelectric speaker used in this study can sustain frequencies up to 20 kHz. Future studies will include testing and characterization of the platform at higher frequency ranges up to 8000 Hz. Additionally, further testing and characterization of the platform will be conducted to include the lowest (60 and 160 Hz) and highest (300 Hz) frequencies commonly measured for males and females at varying sounds volumes.

The in vitro vibratory platform developed in this study is unique in that the wells in our platform experience different magnitudes of displacement (Figure 4). Repeatability of these displacements could prove to be advantageous for future studies involving the study of cellular responses to phonatory regime in different anatomical positions of the vocal fold itself (medially, laterally etc.). To further exploit the sophistication of this platform, various cell types specific to each layer of the vocal tissue could be integrated to explore their response to the vibratory regime. Namely, epithelial cells which can be found in the epithelium of the vocal fold and fibroblasts which are found in the lamina propria of the vocal fold [26]. Understanding the impact that phonation and the associated mechanical stimuli have on the vocal fold microenvironment in different anatomical positions would allow us to better study therapeutic efficacy on injured VF tissue in vitro. Current bioreactors, as shown in the review by Martins et al., were able to incorporate a single cell type at a time [26]. The overall goal is to incorporate multiple cell types simultaneously to mimic the native composition of the VFs more closely. Future studies will include the incorporation of cell culture inserts/dividers within each well to enable multi-culture experiments that will more closely recapitulate the normal histology of native VFs.

Displacements shown in Figure 5 correlate to a magnitude of strain VF tissues experienced during a compression test carried out by Lamprecht et al. [32]. In the study, the authors were able to show a heterogenous spatial distribution of displacement related strain within the vocal fold when deformation was applied with maximums up to 0.5% strain when the vocal fold lamina propria experienced 15 μm of displacement [32]. From this we can draw conclusions about displacement related strain being applied by our vibratory platform to the cells in the wells based on our observed magnitudes of displacement. This information could also be applied to better understand strains that the VF tissue experiences in vivo during the various stages of VF injury.

Supplementary Method S1 explains how we calculated the maximum displacement related strain of the Tegaderm^TM^ membrane during vibration at 100 Hz and 100% volume. Our vibrational platform produced a maximum volumetric strain on the surface of the Tegaderm^TM^ membrane of up to about 0.25% according to our LDV measurements. This is lower than what VF tissues experience in fibrotic regimes when they are generating new collagen and elastin fibers to resist further strain, otherwise known as the breaking point. In a study by Chan et al., the breaking point for healthy VF lamina propria was found to be around 30% strain, which would occur during phonatory regimes that involve singing or shouting [33]. The cells cultured on the surface of the Tegaderm^TM^ membrane in our platform are experiencing a physiologically relevant range of strains since healthy excised porcine VF tissues can experience between 0 and 30% strain before a breaking point is reached [34]. Future studies will include investigating the effect of speaker configuration on the PDMS waveguide with the goal of promoting greater strains representative of fibrotic VF tissue using our vibrational platform.

Bioreactors for VF tissue engineering should incorporate multiple units designed to simultaneously oscillate precise frequencies and displacements. However, current bioreactors have a relatively low number of experimental units with the highest number reported being found in the system developed by Bartlett et al. with 16 units [29]. The in vitro vibratory platform developed in this study incorporates twenty-four experimental units and can be scaled to include commercial plates of six to ninety-six wells. This represents a significant improvement compared to current bioreactors and can enable large scale studies such as testing of multiple biomaterials for VF repair and regeneration, drug response studies, disease modeling, etc. The platform developed in this study will be used in future studies to test various candidate biomaterials for tissue regeneration and repair applications simultaneously in a mechanically relevant environment.

The gene expression of pro-fibrotic and pro-inflammatory genes (ACTA2, MMP1, and HAS1) was evaluated after exposing HVOX and hMSCs to a vibratory regime of 100 Hz at 100% sound volume for 1 or 2 h (Figure 5 and Supplementary Figure S3). The cells were left to rest for 6 h. ACTA2 was selected as a gene of interest due to its crucial role in vocal fold fibrosis. We detected lower ACTA2 expression compared to the static control in most conditions which is contrary to other studies that found ACTA2 expression unchanged in human vocal fold fibroblasts in 2D culture [35,36] and in MSCs in 3D culture [37]. MMPs have a role in controlling the extracellular matrix (ECM) structure and composition by degrading ECM related proteins. When compared to static control, significantly lower MMP1 expression was detected in HVOX at the high displacement range (1 and 2 h of exposure) and in hMSCs at the low displacement range (2 h of exposure). Other studies reported that MMP1 expression was predominantly upregulated in MSCs. For fibroblasts, however, MMP1 expression showed mixed results of being downregulated [38], upregulated [39], or unchanged [35,36,40,41,42]. Lastly, HAS1 expression was evaluated, and no significant changes were detected in either condition for both HVOX and hMSCs. Other studies have reported increased HAS1 expression in 3D cultures involving MSCs [37,43,44,45] and no changes in 2D cultures involving fibroblasts [36]. Overall, it is difficult to make direct comparisons between different studies due to the wide variety in the choice of vibratory regime (frequency, displacement, periodicity, exposure time, etc.), biomaterials, cell types and number, and cell culture formats (2D vs. 3D). Due to its scalability and the potential for multiple displacement ranges, the in vitro vibratory platform developed in this study represents a way to standardize vocal fold bioreactor parameters such as cell type and number, vibratory regime, 2D or 3D culture, biomaterial choice, etc. Future studies will be performed to assess the biological validity of the platform by including a larger set of genes to be analyzed as well as including comparisons between one or more cell types (e.g., fibroblasts, myofibroblasts, epithelial cells, muscle cells, and macrophages) in combination with biomaterials that are currently used in the clinic or in research (e.g., collagen, hyaluronic acid, etc.). Comparisons with data from human and animal models will be performed to further establish the validity of the platform. Ultimately, this approach could serve as a screening tool for novel treatments before preclinical testing.

HVOX cells were also seeded on collagen type I (Supplementary Method S2), a biomaterial commonly used clinically and in vocal fold research, on top of the flexible membrane and the cytocompatibility was evaluated via the live/dead assay (Supplementary Method S3 and Supplementary Figure S4). There were no significant differences in live cell counts between the control group (static culture) and cells exposed to 15, 45, and 105 min of vibration (100 Hz at 100% sound volume). Increased cell death was only observed at 105 min of exposure to vibration. Future studies will include additional cytocompatibility assays for cells (HVOX and hMSCs) cultured for longer periods of time at different vibratory regimes to determine the maximum amount of time that allows for cell survival in the vibratory platform. Overall, these preliminary results are strong indicators that the vibratory platform developed in this study is suitable for in vitro cell based assays.

The in vitro vibratory platform developed in this study has some limitations: (1) the platform does not include more complex forces such as shear, inertial, compressive, or aerodynamic, (2) HVOX used in this study are an immortalized cell line and might respond differently than primary cells, (3) only mono-cultures were investigated at this time, and (4) even though VF healing and fibrosis are very complex events, only a limited number of genes were investigated in this study.

## 5. Conclusions

We designed, built, and characterized a novel, high-throughput, scalable, user-friendly, and tunable in vitro platform for vocal fold tissue engineering applications. The phonatory conditions of the platform were set to incorporate the regular speech fundamental frequencies found in males and females with displacement values going up to 90 μm. The platform can incorporate commercial assay formats ranging from six- to ninety-six-well plates which represents a significant improvement in scalability compared to other available designs. This platform is modular and allows for tunable frequency and displacement regimes. While the platform cannot fully recapitulate the complex microenvironment of the vocal fold, it can act as an alternative strategy to standardize and accelerate tissue engineering studies in the field of vocal fold repair and regeneration.

## Figures and Tables

**Table 1:** The 3D LDV measurement settings.

Measurement Mode	FFT (Frequency Domain)
Averaging	3 Times
Sample Frequency	25 kHz
Bandwidth	0–1.25 kHz
Sample Time	1.28 s
Resolution	781.25 mHz
Speckle Tracking	Best
Vibrometer Controller (Mode)	3D
Vibrometer Velocity	500 mm/s
Vibrometer Tracking Filter	Slow
Number of Scan Points per Well	33 Nodes
Nodal Mesh Radial Density	1.75 mm
Nodal Mesh Angular Density	45 Degrees

**Table 2:** Primer sequences used in RT-qPCR experiments.

Gene	Forward Primer	Reverse Primer
GAPDH	AAGGTGAAGGTCGGAGTCAAC	GGGGTCATTGATGGCAACAATA
ACTA2	CCAGCAGATGTGGATCAGCAAACA	ACGAGTCAGAGCTTTGGCTAGGAA
MMP1	CTCTGGAGTAATGTCACACCTCT	TGTTGGTCCACCTTTCATCTTC
HAS1	GAGCCTCTTCGCGTACCTG	CCTCCTGGTAGGCGGAGAT

## Data Availability

STL files for the 3D printed parts shown in Figure 2 and MP4 videos of the displacement of the piezoelectric speaker in response to 100 Hz 100% input sound volume into and orthogonally to the XY plane are available via this link: https://github.com/andreea-biehl/In-Vitro-Vibratory-Platform-For-Vocal-Fold-Tissue-Engineering-Applications accessed on 10 November 2021.

## Supplementary Materials

The following supporting information can be downloaded at: https://www.mdpi.com/article/10.3390/bioengineering10050602/s1, Figure S1: LDV system setup; Figure S2: LDV measured displacements of piezoelectric speaker; Figure S3: Gene expression profiles; Method S1: Calculation of volumetric strain; Method S2: Hydrogel preparation; Method S3: Live/dead cell viability assay; Figure S4: Cytocompatibility study.
