# Supplementary material for: Scalable and High-Throughput In Vitro Vibratory Platform for Vocal Fold Tissue Engineering Applications
Source: Bioengineering (Basel). 2023 May 17;10(5):602. doi: 10.3390/bioengineering10050602 (PMC10215097; doi:10.3390/bioengineering10050602)
Supplement: Supplementary file 1 [file bioengineering-10-00602-s001.zip › bioengineering-2360607-supplementary.pdf]

bioengineering

Article

Scalable and High-Throughput In Vitro Vibratory Platform for Vocal Fold Tissue Engineering Applications

Andreea Biehl [1,2], Ramair Colmon [1,2], Anastasia Timofeeva [3], Ana Maria Gracioso Martins [1,2], Gregory R. Dion [4], Kara Peters [3] and Donald O. Freytes [1,2,*]

1 Joint Department of Biomedical Engineering, North Carolina State University & University of North Carolina-Chapel Hill, 4130 Engineering Building III, Campus Box 7115, Raleigh, NC 27695, USA; rcolmon@ncsu.edu (R.C.); agracio@ncsu.edu (A.M.G.M.); dofreyte@ncsu.edu

2 Comparative Medicine Institute, North Carolina State University, 1060 William Moore Drive, Raleigh, NC 27606, USA

3 Department of Mechanical and Aerospace Engineering, North Carolina State University, Raleigh, NC 27695, USA; aatimofe@ncsu.edu (A.T.); kjpeters@ncsu.edu (K.P.)

4 Department of Otolaryngology-Head and Neck Surgery, University of Cincinnati, Cincinnati, OH 45267, USA; diongy@ucmail.uc.edu

* Correspondence: dofreyte@ncsu.edu; Tel.: +1-919-513-7933

Supplementary Information

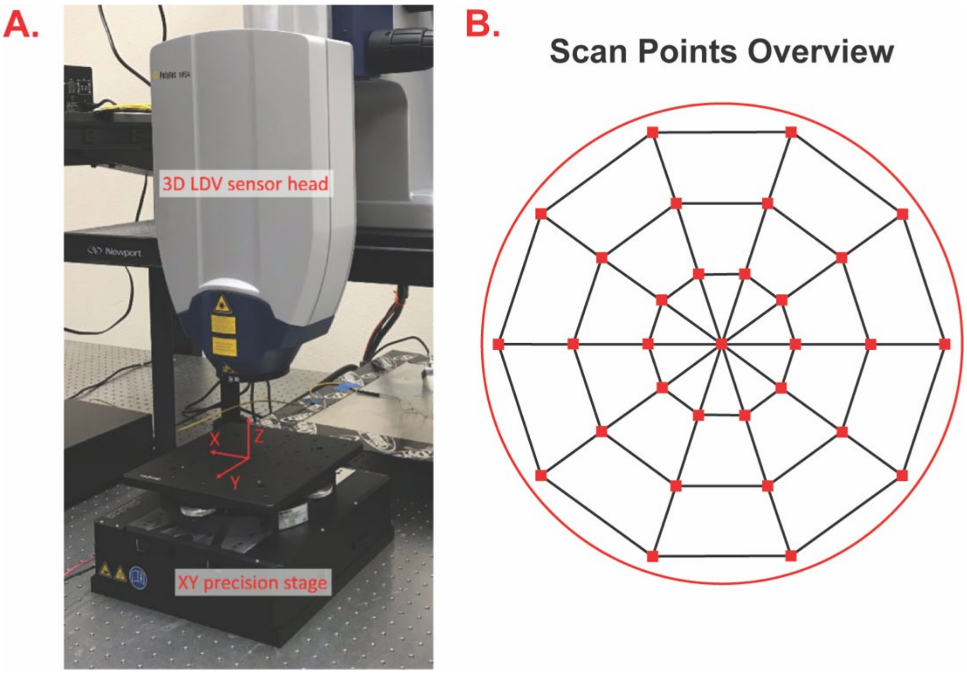

Supplementary Figure S1. LDV system setup. (A) Photograph of the 3D LDV sensor head and XYZ precision stage. (B) Spider web mesh consisting of 33 nodes for each individual well of the 24-well plate.

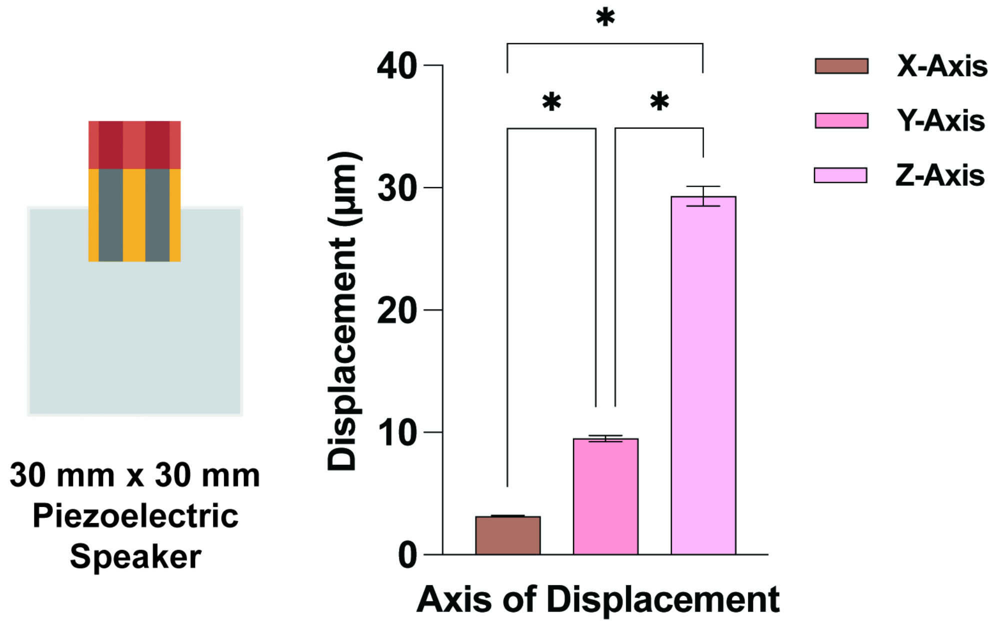

Supplementary Figure S2. (Left) Computer generated schematic of piezoelectric speaker used in speaker characterization with LDV. (Right) LDV measured displacements of Piezoelectric speaker in the X, Y and Z-axes. Bar graphs represent mean ± SEM (standard error of the mean). * = statistically significant ($p < 0.05$). n.s. = not significant ($p > 0.05$).

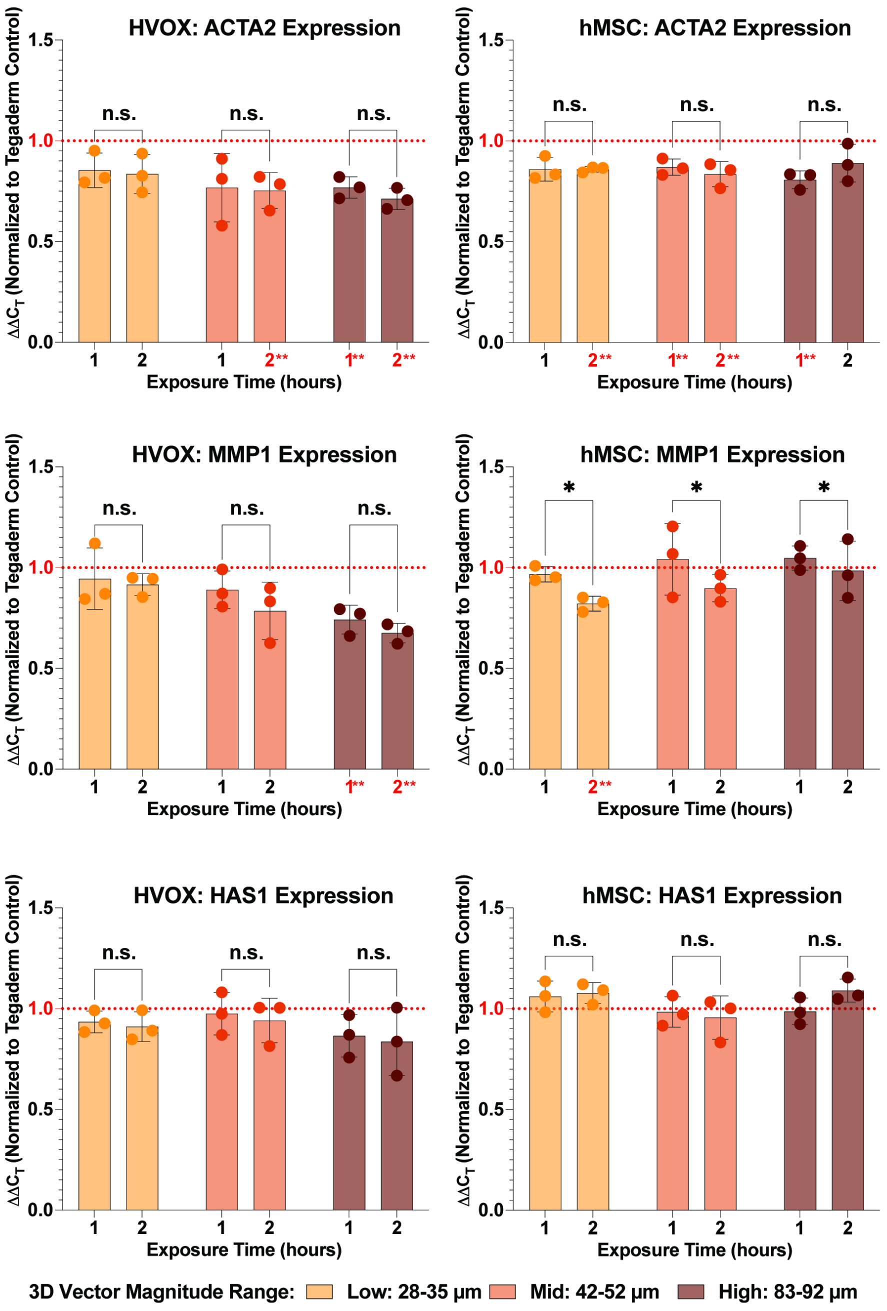

Supplementary Figure S3. Gene expression profiles for ACTA2, MMP1, and HAS1 for HVOX and hMSCs cultured on top of Tegaderm membrane and vibrated at 100 Hz and 100% volume for 1 or 2 hours. Bar graphs represent mean ± SD (standard deviation). Small red asterisks (*) indicate statistical significance relative to the static control normalized to 1.0. Large black asterisks (*) indicate statistical significance relative to or 1- or 2-hour exposure group. n = 3 samples analyzed per experimental group. * = statistically significant ($p < 0.05$). n.s. = not significant ($p > 0.05$).

Supplementary Method S1. Calculation of Volumetric Strain

Volumetric strain was calculated from the resulting LDV data which yielded displacements in the X, Y and Z coordinate axes. To calculate volumetric strain, we defined the original surface area of the Tegaderm™ membrane as our starting area, A. We then added the maximum displacements to our starting area at each node in the spider mesh respectively to define our maximum deformed surface area during vibration at 100 Hz and 100% volume, A′. ΔA is the maximum change in area, mathematically defined by A′-A. We then calculated strain using the following equation, Eq S1:

$$\varepsilon = \frac{\Delta V}{V} = \frac{(\Delta A \cdot T_2)}{A \cdot T_1} = \frac{\Delta A}{A} \quad \text{(S1)}$$

Since the surface of interest is a thin membrane we can consider the thickness, T, to be constant before and after deformation ($T_2 = T_1$). This allows us to simplify our decomposed definition of strain, thus modifying our strain equation to assess the change in Tegaderm™ membrane's surface area after vibration divided by original surface area.

Supplementary Method S2. Hydrogel preparation.

FibriCol I, Collagen Type I > 97% (Advanced Biomatrix, Carlsbad, CA) (Col. I) was used in this study. Col. I hydrogels at a concentration of 6 mg/mL were prepared by adjusting the pH to 7.3 ± 0.2 using 0.1 M NaOH (MilliporeSigma, Burlington, MA) and balancing the salt content using 10X Dulbecco's Phosphate-Buffered Saline (DPBS) (Genesee Scientific, San Diego, CA) and deionized (DI) water. Self-assembly was finalized by placing the solution into the incubator for 45 minutes at 37°C.

Supplementary Method S3. Live/Dead cell viability assay.

Cell viability was assessed using the LIVE/DEAD assay. Briefly, Col. I hydrogels were prepared as described above, pipetted on top of the Tegaderm membrane (0.5 mL/well), and incubated for 45 minutes at 37°C. HVOX were seeded on top of Col. I hydrogels (40,000 cells/well) and allowed to attach overnight. The next day, the cells were exposed to a frequency of 100 Hz at 100% sound volume for 15, 45, and 105 minutes. The samples were stained using the LIVE/DEAD Viability/Cytotoxicity Assay (Life Technologies) according to the manufacturer's instructions. The samples were imaged using fluorescence microscopy (Revolve microscope, Echo, San Diego, CA) and counted using ImageJ (U.S. National Institutes of Health, Bethesda, Maryland).

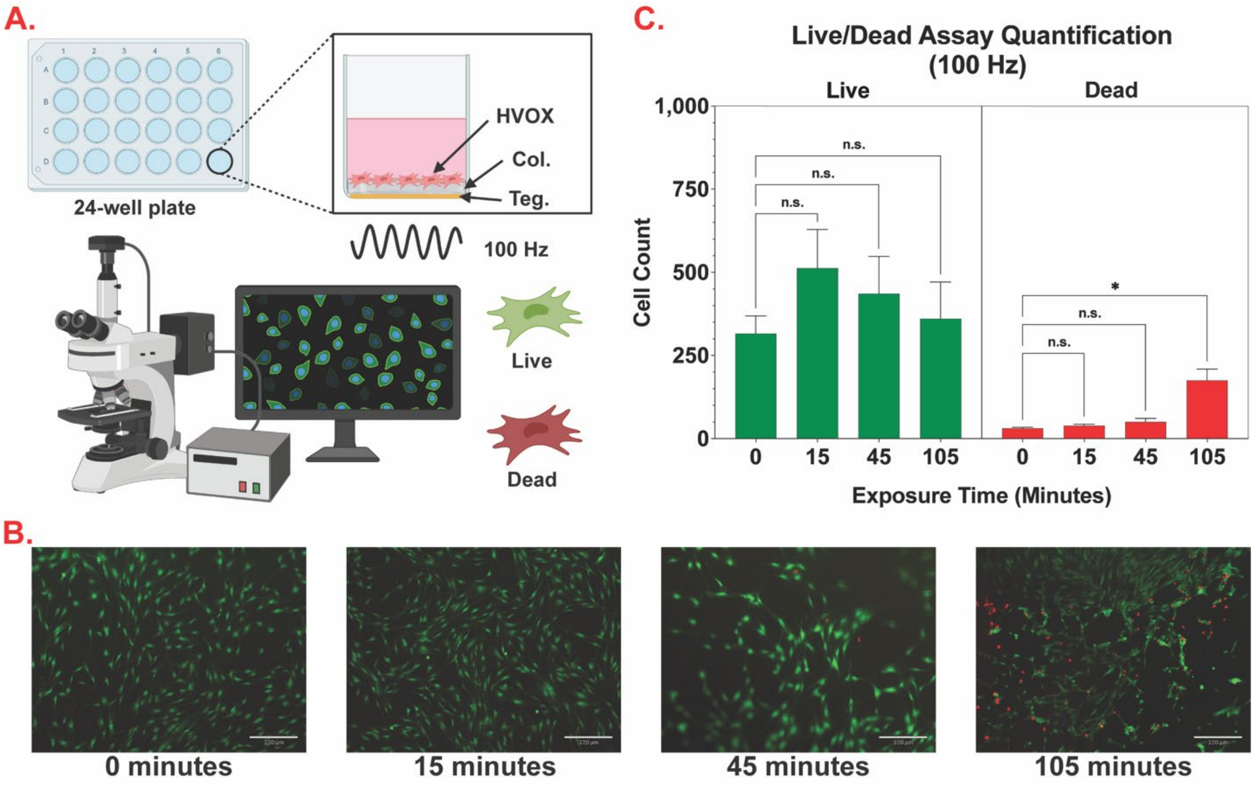

Supplementary Figure S4. Cytocompatibility study. (A) Diagram showing Live/Dead cell viability assay for HVOX grown on Tegaderm (Teg.) membrane with Collagen (Col.) hydrogel. The cells were exposed to a vibrational regime of 100 Hz for 0, 15, 45, and 105 minutes. (B) Representative Live/Dead images for each exposure time. (C) Cell counts (live and dead) using ImageJ (NIH, USA) for each exposure time. The error bars represent the standard error of the mean (SEM). n.s. = not significant. * = statistically significant ($p < 0.05$). n = 3.
